# Supplementary material for: Is it really always only the others who are to blame? GP’s view on medical overuse. A questionnaire study
Source: PLoS One. 2020 Jan 15;15(1):e0227457. doi: 10.1371/journal.pone.0227457 (PMC6961900; doi:10.1371/journal.pone.0227457)
Supplement: S1 File — (DOCX) [file pone.0227457.s001.docx]

| **Fragebogen:**  **“Zu viel, zu wenig oder die falsche Medizin – Was sind Ihre Erfahrungen?”** |
| --- |

Wie hoch schätzen Sie den Anteil an überversorgenden Leistungen in Deutschland aktuell ein?

0% 50% 100%

Wo sehen Sie bezüglich der Versorgungsqualität unserer Patienten aktuell mehr Handlungsbedarf?

Unterversorgung Kein Unterschied Überversorgung

Bitte markieren Sie nach Ihrer persönlichen Einschätzung die drei einflussreichsten Ursachen für Überversorgung.

- Fehlendes Primärarztsystem
- Missachtung von Evidenz/Leitlinien
- Ökonomische Zwänge des Arztes
- Defensive Medizin (medizinische Maßnahmen zur juristischen Absicherung des Arztes)
- Patientenerwartung
- Marketing der Pharmaindustrie
- Fortschritte der Medizintechnik
- Disease mongering ("Krankheitserfindung": Absenkung der Grenzwerte oder Pathologisierung physiologischer Zustände)

Ärztliche Entscheidungen werden von vielen Faktoren bestimmt. Wie sehr stimmen Sie den folgenden Aussagen zu?

|  | Ich stimme überhaupt nicht zu | Ich stimmevoll und ganz zu |
| --- | --- | --- |
| Individuelle Gesundheitsleistungen (IGeL) sind für mich  Überversorgung.. |  |  |
| Umfangreiche Labortests bei Check-Ups erachte ich als sinnvoll, um Einzelfälle besser herauszufiltern. |  |  |
| Besuche eines Pharmavertreters können hilfreich sein, um über neue Produktentwicklungen informiert zu werden. |  |  |
| Patienten mit unspezifischen Kreuzschmerzen sind unzufrieden, wenn ihre Beschwerden nicht mit einer Bildgebung abgeklärt werden. |  |  |
| Je mehr Diagnostik man betreibt, desto mehr Kompetenzen werden einem als Hausarzt zugeschrieben. |  |  |
| Handlungsbereitschaft und Taten stellen Patienten eher zufrieden, als Worte und Abwarten. |  |  |
| Ich sehe es so: „Lieber einmal Überversorgung, als einmal etwas zu übersehen.“ |  |  |
| Arzthaftungsprozesse führen zu Überversorgung, da sich die Ärzte in Folge diagnostisch besser absichern wollen. |  |  |
| Bewertungsportale führen zu Überversorgung, weil Patienten sich so immer mehr als Konsumenten verstehen. |  |  |
| Die angemessene Aufklärung über Nutzen und Schaden von Diagnostik und Therapie scheitert oft am zeitlichen Aufwand. |  |  |
| Leitlinien sind Eingriffe in die professionelle Freiheit der Ärzte, mit dem Ziel Kosten zu dämpfen. |  |  |
| Ich habe mich schon bewusst gegen eine Therapie entschieden, obwohl die neu eingeführte Absenkung des Grenzwertes eine Behandlung nahegelegt hätte. |  |  |
| Ich möchte schnell abklären, was die Ursache für die Symptome meines Patienten ist. |  |  |

Zur Relevanz von Überversorgung gibt es unterschiedliche Meinungen. Welchen Standpunkt vertreten Sie? Entscheiden Sie bitte, ob die folgenden Aussagen aus Ihrer Sicht zutreffen oder nicht.

|  | Trifft überhaupt  nicht zu | Trifft voll und ganz zu |
| --- | --- | --- |
| In Unterhaltungen zwischen Ärzten findet das Thema  Überversorgung kaum Beachtung. |  |  |
| Überversorgung in einem Bereich des Gesundheitswesens führt zu weniger Kapazitäten in anderen. |  |  |
| Unterversorgung von Patienten sollte eher in Angriff genommen werden als deren Überversorgung. |  |  |
| Die Debatte um Überversorgung erscheint mir eher als ein Ablenkungsmanöver für Finanzierungsprobleme im Gesundheitswesen. |  |  |
| Gerade Zufallsbefunde führen dazu, dass sich Diagnostik und  Therapie unnötig in die Höhe schaukeln.. |  |  |
| Ich kenne Patienten, denen ein Schaden aus Überversorgung entstanden ist. |  |  |

Welche Herangehensweise zum Erreichen einer bedarfsgerechten Versorgung erachten Sie für sinnvoll? Entscheiden Sie bitte, ob die folgenden Aussagen aus Ihrer Sicht zutreffen oder nicht.

|  | Trifft überhaupt  nicht zu | Trifft voll und ganz zu |
| --- | --- | --- |
| Als Arzt sollte man mit seinen Patienten über die Kosten von Tests und Medikamenten sprechen. |  |  |
| Um der Überversorgung wirksam zu begegnen, ist mehr Engagement der Politik notwendig. |  |  |
| Ein striktes Primärarztsystem mit dem Hausarzt als „Gatekeeper“ ist gut geeignet, unnötige Diagnostik und Therapie zu verhindern. |  |  |
| Mit steigender Berufserfahrung wird es leichter Überversorgung zu vermeiden. |  |  |
| Ich vertraue darauf, dass die Leitlinien meiner Fachgesellschaft von finanziell unabhängigen Experten entwickelt werden. |  |  |
| Leitlinien sollten explizit darauf hinweisen, gewisse Maßnahmen nicht zu ergreifen. |  |  |

In der Öffentlichkeit beginnt man auf verschiedene Arten Überversorgung zu thematisieren. Von welchen der folgenden Kampagnen haben Sie schon einmal gehört?

- "Choosing wisely"
- "Smarter medicine"
- "Less is more"
- "Klug entscheiden"
- "Quartäre Prävention"
- Keine der genannten Kampagnen

Mit welchen dieser Kampagnen haben Sie sich schon aktiv auseinandergesetzt?

- Keine der genannten Kampagnen
- "Choosing wisely"
- "Smarter medicine"
- "Less is more"
- "Klug entscheiden"
- "Quartäre Prävention"

Die „Choosing Wisely“-Initiative empfiehlt, bestimmte diagnostische und therapeutische Maßnahmen nicht durchzuführen. Wie denken Sie darüber? Entscheiden Sie bitte, ob Sie den folgenden Aussagen zustimmen oder nicht.

|  | Ich stimme  überhaupt nicht zu | Ich stimme voll und ganz zu |
| --- | --- | --- |
| Keine bildgebenden Maßnahmen bei Kreuzschmerzen in den ersten sechs Wochen, es sei denn, Warnsignale wie neurologische Ausfälle oder Osteomyelitis-Verdacht sind vorhanden |  |  |
| Keine jährlichen EKGs oder kardiologischen Screeninguntersuchungen bei asymptomatischen Niedrig-Risiko- Patienten. |  |  |
| Keine DXA-Messung der Knochendichte bei Frauen unter 65  Jahren oder Männern unter 70 Jahren ohne Risikofaktoren. |  |  |
| Kein Skoliosescreening bei asymptomatischen Patienten. |  |  |
| Keine Blut- und Urinuntersuchungen bei asymptomatischen Patienten. |  |  |
| Statin-Generika als Startmedikamente bei Lipidsenkern verwenden. |  |  |
