# Supplementary material for: Is it really always only the others who are to blame? GP’s view on medical overuse. A questionnaire study
Source: PLoS One. 2020 Jan 15;15(1):e0227457. doi: 10.1371/journal.pone.0227457 (PMC6961900; doi:10.1371/journal.pone.0227457)
Supplement: S2 File — (DOCX) [file pone.0227457.s002.docx]

| **Questionnaire:**  **“Too much, too little or the wrong kind of medicine – what are your experiences?”** |
| --- |

How high would you estimate the percentage of medical overuse in Germany at the moment?

0% 50% 100%

Where do you currently see more need for action regarding the quality of treatment for our patients?

medical underuse no difference medical overuse

Please mark - according to your own opinion - the three most influential causes of overtreatment.

The lack of a primary care system

Disregard of evidence/guidelines

Economic pressure on the side of the doctor

Defensive medicine (medical measures in order for the doctor to be legally protected)

Patient expectation

Marketing of the pharmaceutical industry

Advances in medical technology

Disease mongering (“invention of diseases”: lowering of the threshold or pathologising of physiological conditions)

Medical decisions are influenced by a number of factors. To what extent do you agree with the following statements?

|  | I completely  disagree | I completely  agree |
| --- | --- | --- |
| I consider individual health services (IGeL) to be a form of overtreatment. |  |  |
| I consider extensive laboratory tests during check-ups to be useful in order to filter individual cases. |  |  |
| Visits by pharmaceutical representatives can be helpful in order to be informed over new product developments. |  |  |
| Patients with unspecific back pain are dissatisfied if their symptoms are not being checked via imaging processes. |  |  |
| A GP is attributed with more competence if he/she conducts more diagnosis. |  |  |
| Patients are more easily satisfied through actions and the readiness to act than through a “wait and see” approach. |  |  |
| I would rather overuse forms of treatments once than to miss something once. |  |  |
| Liability processes in the medical field lead to overuse since as a result, doctors want to protect themselves in regard to the diagnosis. |  |  |
| Assessment portals lead to medical overuse since as a result patients consider themselves more and more to be consumers. |  |  |
| Temporal expenditure is often the reason for the failure of appropriate information about benefits and harm of diagnosis and therapy. |  |  |
| Guidelines are an infringement of professional liberties of doctors in order to reduce costs. |  |  |
| I have already consciously decided against therapeutic measures, even though the newly implemented lower threshold would have suggested treatment of some form. |  |  |
| I want to figure out the cause for my patient’s symptoms as quickly as possible. |  |  |

Opinions diverge when it comes to the relevance of medical overuse. What is your point of view? Please decide - according to your opinion - whether the following statements are fully correct or not correct at all.

|  | Not correct  at all | Fully  correct |
| --- | --- | --- |
| The topic of medical overuse is mostly overlooked in medical discussions. |  |  |
| Overuse in one field of health care leads to less capacities in a different field. |  |  |
| Underuse of treatment of patients should be tackled rather than overtreatment of such. |  |  |
| The debate surrounding medical overuse seems to be - in my opinion - more of a diversionary tactic to cover up financing problems in the health care system. |  |  |
| Especially incidental findings lead to an unnecessary blowing up of diagnosis and therapy. |  |  |
| I know patients that experienced damage through medical overuse. |  |  |

Which approach do you consider useful in order to achieve a needs-based supply system? Please decide - according to your opinion - whether the following statements are fully correct or not correct at all.

|  | Not correct  at all | Fully  correct |
| --- | --- | --- |
| As a doctor, one should talk to one’s patients about the costs of tests and medication. |  |  |
| More political engagement is necessary in order to counteract medical overuse effectively. |  |  |
| A strict primary doctor system with the GP as “gatekeeper” is useful to prevent unnecessary diagnosis and therapy. |  |  |
| Preventing medical overuse becomes easier with increasing professional experience. |  |  |
| I trust that the guidelines of my specific expert association are developed by financially independent experts. |  |  |
| Guidelines should explicitly point out that certain measures need not be taken. |  |  |

Medical overuse is becoming an increasingly popular theme for discussion in public. Which of the following campaigns have you ever heard of?

Choosing wisely

Smarter medicine

Less is more

Decide wisely

Quaternary prevention

None of these campaigns

Which of these campaigns have you already actively dealt with?

*(Note: Respondents should see only the campaigns they had already heard of.)*

- None of these campaigns
- Choosing wisely
- Smarter medicine
- Less is more
- Decide wisely
- Quaternary prevention

The "Choosing wisely" initiative recommends that certain diagnostic and therapeutic measures should not be carried out. What do you think? Please decide whether you agree or disagree with the following statements.

|  | I completely  disagree | I completely   agree |
| --- | --- | --- |
| Don’t perform imaging for low back pain within the first six weeks unless red flags are present. |  |  |
| Don’t order annual electrocardiography or any other cardiac screening for asymptomatic, low-risk patients. |  |  |
| Don’t use dual energy x-ray absorptiometry to screen for osteoporosis in women younger than 65 years or in men younger than 70 years with no risk factors. |  |  |
| Don’t screen adolescents for scoliosis. |  |  |
| Don’t obtain blood chemistry panels (e.g., basic metabolic panel) or perform urinalyses for screening in asymptomatic, healthy adults. |  |  |
| Use only generic statins when initiating lipid lowering drug therapy. |  |  |
