# Supplementary material for: Is it really always only the others who are to blame? GP’s view on medical overuse. A questionnaire study
Source: PLoS One. 2020 Jan 15;15(1):e0227457. doi: 10.1371/journal.pone.0227457 (PMC6961900; doi:10.1371/journal.pone.0227457)
Supplement: Attachment — Submitted filename: PLOS Review.docx [file pone.0227457.s004.docx]

**PLOS Review – Medical Overuse, August 2019**

The article is on an important topic area—overuse of medical services.

The article is written clearly, and the tables are well designed.

Overall, the authors followed a reasonable study design was followed. They used sound methods for developing and testing their questionnaire. The used a registry of general practitioners (GPs) for sample selection. I was curious about why the authors limited their sample to GPs, as they noted that in Germany, unlike other countries, GPs do not serve as gatekeepers for access to medical specialists.

The study has two substantial methodological issues that limit the utility of the results. The authors followed sound survey procedures, with the exception of survey fielding. They only allowed 18 days for GPs to return the survey and sent out a single reminder. A longer data collection period and more follow-up probably would have improved the response rate.

Selection Bias. The study had a good sample plan, but only 18% of subjects returned complete questionnaires. An 18% response rate almost certainly entails some sort of selection bias. The authors mention that only respondents who are interested in the topic of medical overuse may have responded to the survey, although there is almost no way to test that. The authors report demographics for the physicians who returned the survey. I don’t know if they have access to demographics for the entire population of physicians in the geographic area, but if so, it would be helpful to compare the demographics of those who returned the survey with the entire population. While demographics would not tell much of a story about self-selection, it would be beneficial to know if there was selection by age or some other characteristic. In particular, since the authors found that younger doctors viewed medical overuse as more of a problem than older doctors, information on age of the sample compared with age of the population of GPs would have bolstered results.

Inefficient use of data. The questionnaire was comprised mainly of Likert-style questions on a scale of 1 to 6. The authors dichotomized responses into positive and negative. They provide no explanation about why they chose to do this, with the result that they lost information.
